# Association of genetic liability for psychiatric disorders with accelerometer-assessed physical activity in the UK Biobank

**DOI:** 10.1371/journal.pone.0249189

**Published:** 2021-03-26

**Authors:** Charlotte A. Dennison, Sophie E. Legge, Matthew Bracher-Smith, Georgina Menzies, Valentina Escott-Price, Daniel J. Smith, Aiden R. Doherty, Michael J. Owen, Michael C. O’Donovan, James T. R. Walters

**Affiliations:** 1 MRC Centre for Neuropsychiatric Genetics and Genomics, Division of Psychiatry and Clinical Neurosciences, School of Medicine, Cardiff University, Cardiff, United Kingdom; 2 School of Biosciences, Dementia Research Institute, Cardiff University, Cardiff, United Kingdom; 3 Division of Psychiatry and Clinical Neurosciences, School of Medicine, Dementia Research Institute, Cardiff University, Cardiff, United Kingdom; 4 Institute of Health and Wellbeing, University of Glasgow, Glasgow, United Kingdom; 5 Big Data Institute, Li Ka Shing Centre for Health Information and Discovery, University of Oxford, Oxford, United Kingdom; 6 National Institute of Health Research Oxford Biomedical Research Centre, Oxford, United Kingdom; Department of Psychiatry and Neuropsychology, Maastricht University Medical Center, NETHERLANDS

## Abstract

Levels of activity are often affected in psychiatric disorders and can be core symptoms of illness. Advances in technology now allow the accurate assessment of activity levels but it remains unclear whether alterations in activity arise from shared risk factors for developing psychiatric disorders, such as genetics, or are better explained as consequences of the disorders and their associated factors. We aimed to examine objectively-measured physical activity in individuals with psychiatric disorders, and assess the role of genetic liability for psychiatric disorders on physical activity. Accelerometer data were available on 95,529 UK Biobank participants, including measures of overall mean activity and minutes per day of moderate activity, walking, sedentary activity, and sleep. Linear regressions measured associations between psychiatric diagnosis and activity levels, and polygenic risk scores (PRS) for psychiatric disorders and activity levels. Genetic correlations were calculated between psychiatric disorders and different types of activity. Having a diagnosis of schizophrenia, bipolar disorder, depression, or autism spectrum disorders (ASD) was associated with reduced overall activity compared to unaffected controls. In individuals without a psychiatric disorder, reduced overall activity levels were associated with PRS for schizophrenia, depression, and ASD. ADHD PRS was associated with increased overall activity. Genetic correlations were consistent with PRS findings. Variation in physical activity is an important feature across psychiatric disorders. Whilst levels of activity are associated with genetic liability to psychiatric disorders to a very limited extent, the substantial differences in activity levels in those with psychiatric disorders most likely arise as a consequences of disorder-related factors.

## Introduction

Estimates suggest that physical inactivity causes 9% of premature mortality and 6–10% of the major non-communicable diseases worldwide [1]. Research consistently shows that individuals who engage in less physical activity report more stress [2], perform worse on cognitive tasks [3], are more likely to become depressed [4, 5], and are at increased risk of cardiovascular disease, cancer, hypertension and diabetes [6]. Physical activity can be a core indicator of mental illness, from the increased activity seen in attention deficit hyperactivity disorder (ADHD) and mania to the decreased activity seen in depression and as part of the negative symptoms of schizophrenia [7–10]. Understanding the factors contributing to physical activity may assist in improving mental health, as well as reducing risk of chronic physical health conditions, many of which are known to be increased in individuals with severe mental illness [11].

Studies of physical activity have predominantly relied on self-report measures but this may lead to unreliable estimates of activity, especially for those with mental illness. A recent study reported marked differences when comparing accelerometer-measured activity between individuals with schizophrenia and controls, but not for self-reported activity [12]. This suggests that objective measures may better characterise physical activity in individuals with mental health disorders, and such approaches are being considered as part of clinical psychiatric care [13]. Research using accelerometers in the UK Biobank has shown physical activity to be a polygenic trait, with a heritability of around 23% in women and 20% in men [14].

Physical activity in those with psychiatric disorders may be influenced by genetic liability to activity in the wider population, although it may primarily reflect disorder-specific factors. Conversely, it is possible that genetic liability for psychiatric disorders influences physical activity, rather than activity differences being a consequence of the illness. Other factors known to be associated with both psychiatric disorders and physical activity, such as smoking, obesity, and social deprivation [15–17], may also confound this relationship and thus require investigation.

A small number of studies have applied Mendelian Randomisation (MR) methodology [18] to examine the hypothesis that low physical activity might be a risk factor for developing psychiatric disorders, particularly depression and schizophrenia [19, 20]. Some have reported findings consistent with the hypothesis that low physical activity might be causally related to depression, although one of the limitations of such studies is the lack of robust genetic instruments for MR analyses. Currently, the relationship between genetic liability to physical activity and psychiatric disorders is unclear.

We aimed to assess the levels of objectively-measured physical activity in individuals with psychiatric disorders, and establish whether genetic liability for psychiatric disorders is associated with levels of physical activity in a population sample. We hypothesised that objective levels of physical activity in individuals with mental health disorders would differ from individuals without, and that there would be an association between polygenic risk for psychiatric disorders and level of physical activity in individuals without mental health disorders.

## Method

### Participants

Study participants were from the population-based UK Biobank sample, a national cohort study of over 500,000 individuals aged 40–69 at the time of recruitment from one of 22 assessment centres across the UK between 2006–2010 [21]. Between 2013 and 2015, a subset of individuals was invited to participate in a study of device-measured physical activity (see S1 Fig for timeline of data collection). A random group of participants with a valid email address were invited, with the exception of those residing in the North West region, who were excluded due to concerns over participant burden. Of the 236,519 individuals approached, 106,053 consented to participate [22]. Our study was conducted as part of UK Biobank project number 13310. Ethical approval for UK Biobank was granted by the North West Multi-Centre Ethics Committee and all participants provided written informed consent. All procedures contributing to this work comply with the ethical standards of the relevant national and institutional committees on human experimentation and with the Helsinki Declaration of 1975, as revised in 2008.

### Psychiatric diagnosis

A diagnosis of schizophrenia, bipolar disorder, depression, ADHD, or an autism spectrum disorder (ASD) was defined from (i) self-report at the initial assessment, (ii) the mental health questionnaire [23] (Field ID: 20544), (iii) a diagnosis recorded on a hospital record, or (iv) a diagnosis recorded on a death record. Where individuals reported multiple diagnoses, they were included in each appropriate diagnostic group. For hospital and death records, participants were deemed to have a diagnosis when the following ICD-10 codes were present: F20 for schizophrenia, F30 or F31 for bipolar disorder, F32 or F33 for depression, F84 for ASD, and F90 for ADHD. We selected these disorders as altered activity can be a prominent feature of the disorder and substantial GWAS data exist to allow us to test genetic hypotheses.

### Genetic data

Genetic data were made available through UK Biobank, following imputation and quality control procedures that have been described elsewhere [24]. Participants were assayed at the Affymetrix Research Services laboratory using the UK Biobank Axiom or UK BiLEVE Axiom purpose-built arrays and imputation was performed using the Haplotype Reference Consortium panel [25]. Subsequent to imputation, we applied further quality control filters to select high-quality SNPs: minor allele frequency > 0.01, imputation score > 0.8, missingness < 0.05, and Hardy-Weinberg equilibrium p-value > 1x10^-6^. All genetic analyses were restricted to participants of European ancestry, confirmed through self-reported data and principal components [24] (UK Biobank Field IDs: 21000, 22009) as described in a previous study [26], and related individuals with a kinship score greater than 0.125 were removed at random.

### Genetic correlations

We measured genetic correlations to examine the relationships between psychiatric disorder genetic risk and five classes of activity: overall, moderate, walking, sedentary, and sleep duration. We used Linkage Disequilibrium (LD) score regression [27, 28] to calculate genetic correlations between each activity class and schizophrenia [29], bipolar disorder [30], major depressive disorder (MDD) [31], ADHD [32], and ASD [33].

### Polygenic risk scores

PRSice [34] was used to derive polygenic risk scores (PRS) for schizophrenia, bipolar disorder, MDD, ADHD, and ASD from the same external discovery datasets used for the genetic correlations, following the methods used by the Psychiatric Genomics Consortium (PGC) [26, 35]. PRS were calculated at six thresholds: p<5x10^-8^, 5x10^-6^, 5x10^-4^, 0.05, 0.1, 0.5. PRS were then standardised as Z-scores for each disorder, to allow for comparison between disorders.

### Accelerometer-measured physical activity

Participants wore an Axivity AX3 tri-axial accelerometer for one week on the wrist of their dominant hand. The accelerometer captures activity at 100Hz with a dynamic range of ±8g. Data were processed by the accelerometer working group [22] in line with standard protocols, including calibration of devices, low-pass filtering to remove high frequency noise, and identification of non-wear periods [22, 36–38]. A measure of overall physical activity was computed by partitioning the data into five second epochs and calculating the mean vector magnitude of each epoch to derive an overall mean acceleration (UK Biobank Field ID: 90012).

We also used derived measures of time spent in sleep and physical activity behaviours. A recent study by Doherty et al. [14] classified the accelerometer activity being undertaken as sedentary, walking, moderate, or sleeping. In this study, participants in an independent sample wore wrist-worn accelerometers and a wearable camera for one week, in order to map the accelerometer readings to an observable activity. Machine learning methods were then used to create a model that was able to classify accelerometer readings into a pre-defined type of activity [14]. The researchers used these data to derive an overall probability of each participant engaging in each type of activity at any given time (Return ID: 1942). Overall level activity was standardised as a Z-score; moderate, walking, sedentary, and sleep were converted into minutes spent per day engaging in the activity type. Individuals with insufficient device wear time, poor device calibration and an overall mean activity Z score greater than 3 were excluded from analysis.

Doherty et al. [14] conducted a GWAS on each of the activity subtypes; we used the summary statistics from these GWAS for LD score regression.

### Analysis

All analyses were corrected for multiple comparisons using false discovery rate (FDR) at p<0.05.

Linear regressions were conducted to measure the effect of diagnosis of schizophrenia, bipolar disorder, depression, ADHD and ASD on all types of activity: overall, moderate, walking, sedentary and sleep. Age, sex, and BMI were included as covariates in all models.

Linear regressions were used to measure the associations between each disorder PRS and each type of activity, with age, sex, BMI, and genotyping array included as covariates in all PRS models. We routinely included the first five principal components as covariates. We also examined 20 principal components for each type of activity, and included any that were significantly associated with the relevant activity. This varied for each test and ranged from one additional component for overall activity, to three additional components each for moderate and sedentary activity.

PRS associations were conducted at the p < .05 threshold for the primary analyses; the remaining five thresholds were also tested for robustness. Individuals with a psychotic disorder, bipolar disorder, depression, ADHD or ASD diagnosis were excluded from all PRS analyses, leaving a total of 76,409 participants. None of the genome-wide association study (GWAS) discovery datasets we have used included the UK Biobank as a sample, but we are unable to definitively exclude an incidental degree of sample overlap. We note however that as calculated here, genetic correlations derived from LD-score regression are robust to sample overlaps [28]. Moreover, all of the intercepts for our genetic correlations are below one, which is consistent with minimal sample overlap between the discovery GWAS and the target sample for PRS.

In order to assess whether the relationship between PRS for psychiatric disorders and physical activity could be influenced by other confounders, we added alcohol use (Field ID: 20414), cannabis use (Field ID: 20453), substance or behavioural addiction (Field ID: 20401), smoking status (Field ID: 20116), fluid intelligence (Field ID: 20016), and Townsend deprivation index (Field ID: 189) as additional covariates in the overall levels of activity PRS models. These data were available on 15,285 participants who had also completed the mental health questionnaire.

To assess whether including BMI as a covariate could be a source of collider bias, we repeated the diagnosis and PRS analyses with BMI excluded from the model.

## Results

Age, sex, and diagnosis were available on 236,502 individuals who were invited to participate in the accelerometer study. Participation in the accelerometer study was significantly, yet minimally, associated with age at recruitment (OR = 1.002; 95% CI = 1.001, 1.003; p = 2.7x10^-6^), as well as with female sex (OR = 1.14; 95% CI = 1.13, 1.16; p = 4.7x10^-59^). Individuals with a diagnosis of schizophrenia, bipolar disorder, depression, ADHD, or ASD were significantly less likely to participate than individuals without a mental health disorder (OR = 0.95; 95% CI = 0.92, 0.98; p = 0.002).

A total of 95,744 participants were included in the study with high quality accelerometer data (56.4% female, mean age at recruitment [SD] 56.2 [7.8], see S2 Fig). 6,527 individuals were classified as having depression, 466 with bipolar disorder, 95 with schizophrenia, 87 with ASD, and 53 with ADHD.

### Activity levels in psychiatric disorders

**Fig 1:**
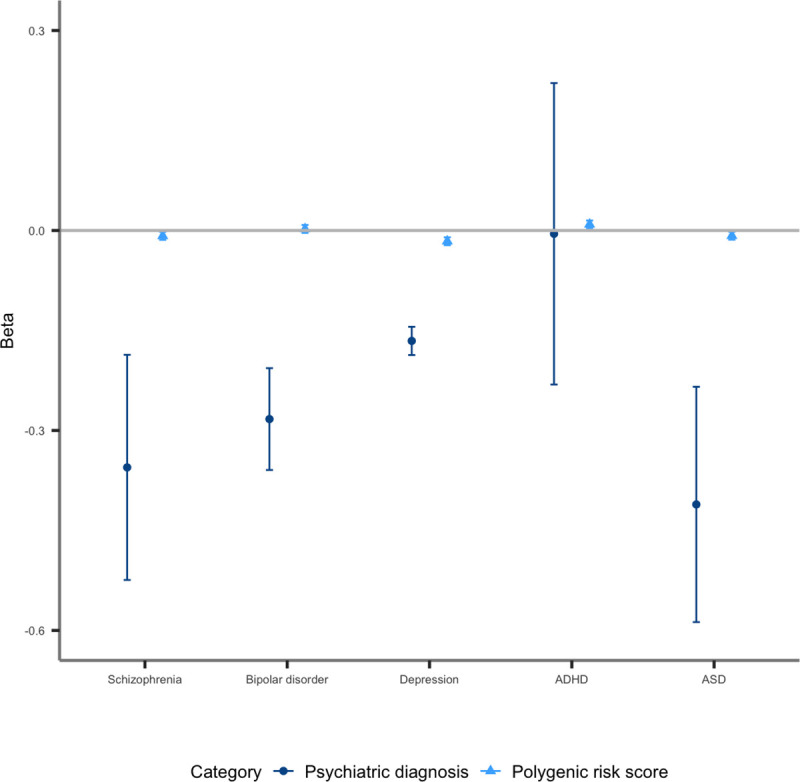
The effect size (beta) for associations between overall activity and diagnoses of, and PRS for, each psychiatric disorder. Error bars indicate 95% confidence intervals. A beta of 1 is equivalent to a 1 standard deviation (SD) change in level of activity between individuals with and without a psychiatric disorder or per 1 SD increase in PRS. We excluded individuals with a psychiatric disorder for PRS analyses.

**Fig 2:**
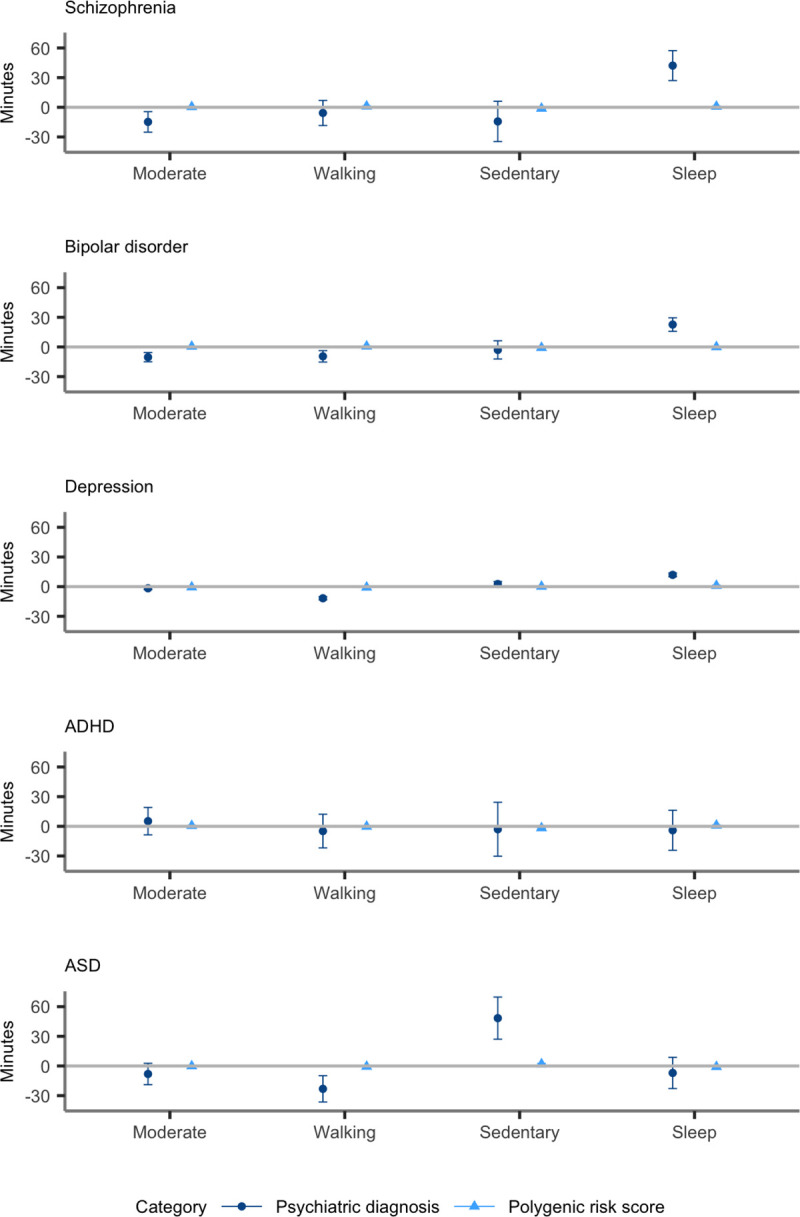
Associations between type of activity in minutes and diagnoses of, and PRS for, each psychiatric disorder. Error bars indicate 95% confidence intervals. A beta of 1 is equivalent to a 1 standard deviation (SD) change in level of activity between individuals with and without a psychiatric disorder or per 1 SD increase in PRS. We excluded individuals with a psychiatric disorder for PRS analyses.

Results are presented in Table 1, Figs 1 and 2. After correcting for multiple comparisons, schizophrenia was associated with reduced levels of overall activity, reduced time spent in moderate activity and longer sleep duration. Bipolar disorder was associated with reduced levels of overall activity, reduced time spent in moderate activity and walking, and longer sleep duration. Depression was associated with reduced levels of overall activity, reduced time spent in moderate activity and walking, and longer sleep duration. ASD was associated with reduced levels of overall activity, reduced time spent walking, and increased time spent in sedentary activity. ADHD was not associated with changes in any type of activity.

**Table 1:** Association between activity and both psychiatric disorders and polygenic risk scores for psychiatric disorders in the UK Biobank.

Disorder	Activity	Psychiatric disorder		Polygenic risk score	
		Beta (95% CI)	P-value	Beta (95% CI)	P-value
Schizophrenia	Overall	-0.4 (-0.5, -0.2)	9.3x10^-5^	-0.01 (-0.01, -0.002)	0.01
	Moderate	-14.8 (-25.1, -4.4)	0.01	0.4 (0.03, 0.8)	0.04
	Walking	-5.8 (-18.5, 7.0)	0.50	1.0 (0.5, 1.5)	9.6x10^-5^
	Sedentary	-14.3 (-34.7, 6.1)	0.25	-1.2 (-2.0, -0.5)	3.0x10^-3^
	Sleep	42.2 (27.1, 57.3)	1.9x10^-7^	0.8 (0.3, 1.3)	0.01
Bipolar disorder	Overall	-0.3 (-0.4, -0.2)	2.5x10^-12^	0.002 (-0.004, 0.01)	0.51
	Moderate	-10.3 (-15.0, -5.7)	4.3x10^-5^	0.5 (0.1, 0.8)	0.02
	Walking	-9.5 (-15.2, -3.7)	3.0x10^-3^	0.7 (0.2, 1.1)	0.01
	Sedentary	-3.0 (-12.2, 6.3)	0.63	-0.8 (-1.5, -0.1)	0.04
	Sleep	22.7 (15.8, 29.5)	4.3x10^-10^	-0.01 (-0.5, 0.5)	0.96
Depression	Overall	-0.2 (-0.2, -0.1)	1.5x10^-51^	-0.02 (-0.02, -0.01)	2.1x10^-6^
	Moderate	-1.7 (-3.0, -0.4)	0.02	-0.6 (-0.9, -0.2)	0.01
	Walking	-11.8 (-13.4, -10.2)	4.0x10^-46^	-0.8 (-1.2, -0.3)	3.0x10^-3^
	Sedentary	2.6 (0.1, 5.2)	0.07	0.1 (-0.6, 0.8)	0.83
	Sleep	11.9 (10.0, 13.8)	8.4x10^-34^	1.0 (0.4, 1.5)	1.8x10^-3^
ADHD	Overall	0.01 (-0.2, 0.2)	0.97	0.01 (0.003, 0.02)	0.01
	Moderate	5.2 (-8.7, 19.1)	0.58	0.6 (0.2, 0.9)	0.01
	Walking	-4.8 (-21.8, 12.2)	0.66	-0.2 (-0.7, 0.2)	0.39
	Sedentary	-3.0 (-30.3, 24.3)	0.87	-1.7 (-2.5, -1.0)	1.4x10^-5^
	Sleep	-4.0 (-24.3, 16.2)	0.76	0.9 (0.4, 1.4)	3.0x10^-3^
ASD	Overall	-0.4 (-0.6, -0.2)	1.8x10^-5^	-0.01 (-0.01, -0.002)	0.01
	Moderate	-8.1 (-18.9, 2.7)	0.22	-0.1 (-0.5, 0.2)	0.54
	Walking	-23.1 (-36.4, -9.9)	1.0x10^-3^	-0.6 (-1.0, -0.1)	0.02
	Sedentary	48.4 (27.1, 69.7)	2.7x10^-5^	1.9 (1.2, 2.6)	2.5x10^-6^
	Sleep	-7.1 (-22.9, 8.7)	0.50	-0.8 (-1.4, -0.3)	0.01

Columns represent the disorder, type of activity, effect size (beta), 95% confidence intervals, and FDR-corrected p-value of the association between either a diagnosis of the disorder or PRS for the disorder and level of activity. Effect size for overall activity corresponds to standard deviation change in activity, effect sizes for all other types of activity correspond to minutes per day of activity. We excluded individuals with a psychiatric disorder for PRS analyses.

#### Genetic correlations

**Fig 3:**
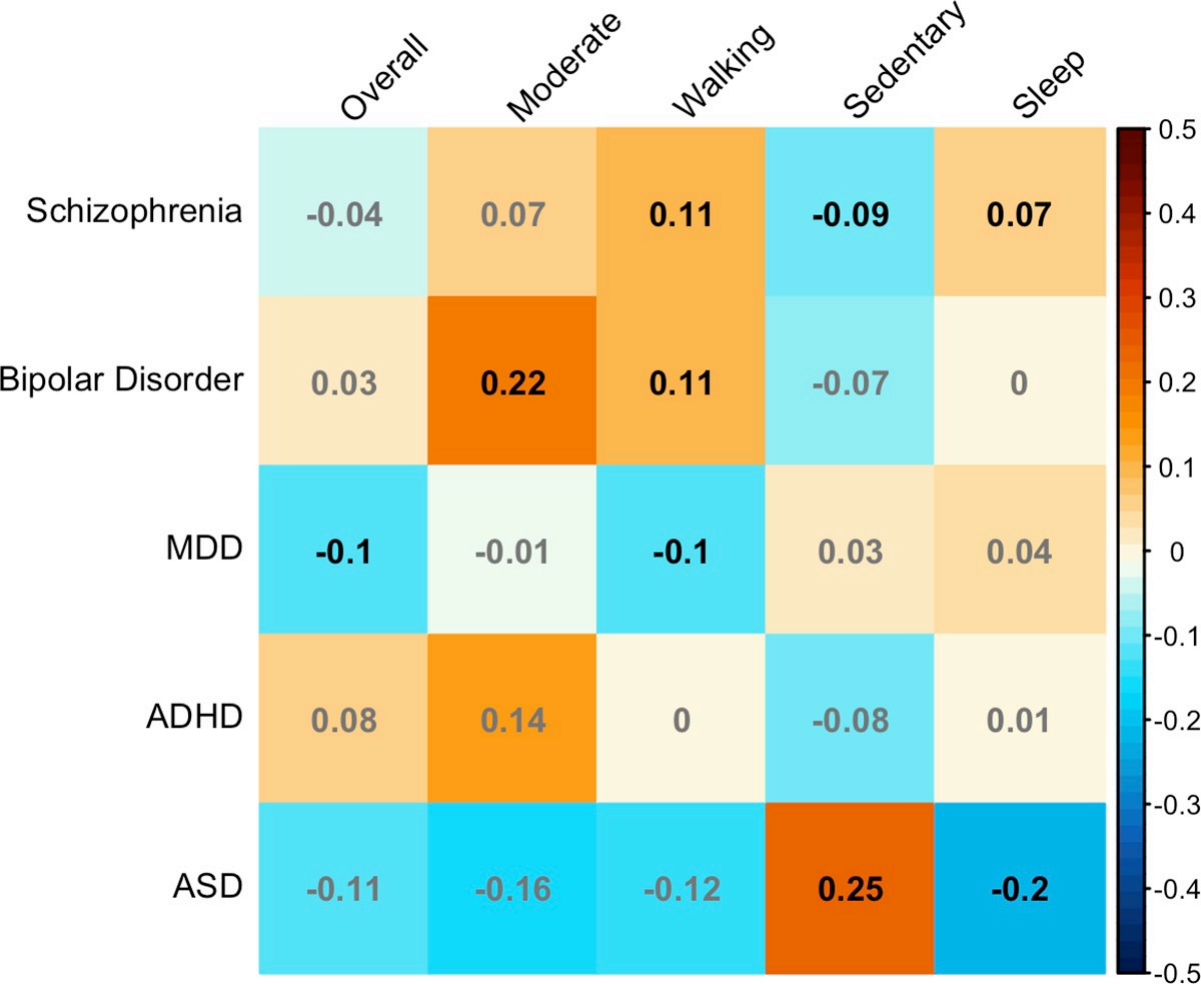
Genetic correlation matrix displaying the correlation coefficient (r_g_), with square colour indicating direction of effect. Black text indicates correlation coefficients significant at FDR-corrected p < .05, grey text indicates non-significant correlations.

Schizophrenia was genetically correlated with more *time spent walking* (r_g_ = 0.11, p = 0.01), reduced *time spent in a sedentary activity* (r_g_ = -0.09, p = 0.02), and longer *sleep duration* (r_g_ = 0.07, p = 0.04). Bipolar disorder was genetically correlated with greater *moderate activity* (r_g_ = 0.22, p = 4x10^-3^) and more *time spent walking* (r_g_ = 0.11, p = 0.05). MDD was genetically correlated with reduced *overall activity* (r_g_ = -0.1, p = 0.01) and reduced *walking* (r_g_ = -0.1, p = 0.02). ASD was significantly genetically correlated with greater *sedentary activity* (r_g_ = 0.25, p = 3x10^-3^) and reduced *sleep duration* (r_g_ = -0.2, p = 0.01). Regression coefficients for all comparisons are displayed in Fig 3 and full results are presented in S1 Table.

### Polygenic risk scores

Results are presented in Table 1, Figs 1 and 2. In individuals without a mental health disorder, after correcting for multiple comparisons, schizophrenia PRS was associated with reduced levels of overall activity, increased time spent in moderate activity, increased time spent walking, reduced time spent in sedentary activity, and longer sleep duration. Bipolar disorder PRS was associated with increased time spent in moderate activity, increased time spent walking, and reduced time spent in sedentary activity. MDD PRS was associated with reduced levels of overall activity, reduced time spent in moderate activity, reduced time spent walking, and longer sleep duration. ADHD PRS was associated with increased levels of overall activity, increased time spent in moderate activity, reduced time spent in sedentary activity, and longer sleep duration. ASD PRS was associated with reduced levels of overall activity, reduced time spent walking, increased time spent in sedentary activity, and shorter duration of sleep.

PRS associations were consistent across the thresholds tested (S2–S6 Tables). The proportion of variance explained by PRS for each type of activity are demonstrated in S3 Fig. The effect sizes for schizophrenia, bipolar disorder, and ADHD PRS in association with overall levels of activity remained consistent after covarying for alcohol use, cannabis use, substance or behavioural addiction, smoking status, fluid intelligence, and Townsend deprivation index. However, effect sizes were reduced for MDD and ASD PRS when these covariates were included (S7 Table). Results were consistent between models that included and excluded BMI as a covariate (S8 Table).

## Discussion

In this study, we found levels of objectively-measured physical activity to be altered in UK Biobank participants with a diagnosis of schizophrenia, bipolar disorder, depression, or ASD. We observed several significant genetic correlations between psychiatric disorders and types of activity, suggesting that the genetic architecture of physical activity is shared, to a small extent, with the genetic architecture of psychiatric disorders. Increased PRS for schizophrenia, bipolar disorder, MDD, ADHD, and ASD were associated with significant, yet minimal, changes in levels of physical activity in individuals without mental health disorders.

### Activity levels in psychiatric disorders

We found that individuals with schizophrenia, bipolar disorder, depression and ASD had reduced levels of overall physical activity in comparison to individuals without a mental health diagnosis, consistent with previous research demonstrating reduced levels of subjectively-measured activity in psychiatric disorders [7–9, 39]. Our results expand upon existing findings by demonstrating these effects in a sample that is larger than has been reported previously, and by using objective methods to capture activity. Additionally, we present novel findings that individuals with psychiatric disorders show different patterns of activity, including reduced moderate activity and longer sleep duration in individuals with schizophrenia, bipolar disorder, and depression, and increased levels of sedentary activity in individuals with depression and ASD. Previous research has suggested that disruption to circadian rhythm, measured by smaller differences between the most active and least active periods of the day, is associated with increased likelihood of depression and bipolar disorder [39]. Our results support and extend these findings by demonstrating a reduced pattern of activity with longer sleep duration in individuals with these disorders. The disruption in physical activity observed in this study could arise either from disorder-related factors such as symptoms or medication side-effects, or from risk factors for the disorders. Together these findings suggest that disrupted activity may be an important aspect of psychiatric illness.

ADHD diagnosis, however, was not associated with level of physical activity. Between 40 and 60% of children with ADHD continue to show symptoms in adulthood [40], thus it is possible that many participants with an ADHD diagnosis may be asymptomatic by the time of data collection. Alternatively, our result may reflect the fact that the UK Biobank is a cohort of individuals aged over 40, who may be less likely to have received a diagnosis of ADHD due to changes in the awareness of the disorder over time [41].

### Genetic liability for psychiatric disorders

In individuals without a mental health disorder, genetic liability for schizophrenia, MDD, ADHD, and ASD was significantly associated with the overall level of physical activity. We also observed several associations between PRS and levels of subtypes of activity, including greater levels of moderate activity with increased PRS for schizophrenia, bipolar disorder, and ADHD. These findings are consistent with the hypothesis that physical activity is one of the behaviours that is influenced by genetic risk for psychiatric disorders in individuals without mental health disorders. However, both the estimated effect sizes of PRS associations and the genetic correlations were small, suggesting that the differences we observed in levels of activity in individuals with psychiatric disorders are the result of manifesting the disorders *per se*, rather than reflections of genetic vulnerability to them. Such factors may be secondary, for example the use of psychotropic medication [42] or could be symptoms of the disorder itself. The associations between overall levels of activity and MDD and ASD PRS were attenuated after controlling for alcohol use, cannabis use, substance addiction, smoking, cognition, and deprivation, further suggesting that non-genetic factors may have a stronger effect on levels of activity than PRS. This has important consequences for the physical health of those with psychiatric disorders, particularly as these individuals are known to be at greater risk of numerous physical health conditions [11] and early mortality [43], which may in part be due to reduced levels of activity. Further research aiming to understand why physical activity is affected in psychiatric disorders is necessary to address and improve physical and mental health outcomes of individuals with these disorders.

### Strengths and limitations

We were unable to exclude the possibility of an overlap of participants between training and target samples when calculating PRS. However, the genetic correlation analysis, for which non-overlapping samples is not a requirement, supports our PRS findings. Case overlap between the discovery GWAS and our target sample (UK Biobank) would lead to overestimation of the PRS effect sizes for any PRS associations that are driven by psychiatric diagnoses. As the effect sizes we observed are already trivial, any overestimation in effect size would not in any important way alter the conclusions. A strength of our study is the substantial sample size, allowing greater power to detect small genetic effects. However, it is important to note that our sample is a population-based cohort and the limited number of psychiatric cases within UK Biobank means we were under-powered to measure the influence of genetic risk in those with psychiatric illness. Individuals with mental health disorders are known to be under-represented in the UK Biobank and those that are included tend to be a more highly functioning group than people with the disorders as a whole [44]. Thus, the differences in levels of activity in individuals with psychiatric disorders are likely to be underestimated in our study.

Obtaining accelerometer data in a sufficiently-powered sample of individuals with psychiatric disorders is a notable challenge. Nevertheless, future research would benefit from the study of objectively-measured activity in individuals with mental health disorders, particularly given findings demonstrating substantial discrepancies between self-report and accelerometer-measured activity [12].

## Conclusions

We found novel evidence of association between schizophrenia, MDD, ADHD, and ASD PRS and accelerometer-assessed physical activity in the UK Biobank. Levels of physical activity were significantly reduced in UK Biobank participants with a diagnosis of schizophrenia, bipolar disorder, depression and ASD, emphasising the need for clinical intervention to address levels of physical activity in these populations. Furthermore, several significant genetic correlations were observed with subtypes of physical activity, most notably between ASD and sedentary activity, ASD and sleep duration, and bipolar disorder and moderate activity. Overall, our findings indicate a weak to modest sharing of liability to the psychiatric disorders and types of activity we have tested, suggesting that the much more substantial differences in levels of activity seen in individuals with the psychiatric disorders are mainly consequences of the disorders, rather than reflections of liability to them.

## Supporting information

S1 Fig Timeline of data collection. (PDF)

S2 Fig Flow diagram of exclusion criteria. (PDF)

S3 Fig Variance explained by polygenic risk scores. Proportion of variance (R^2^) explained by each polygenic score threshold, relative to a baseline model including only covariates, for (A) overall activity, and (B) specific types of activity. Asterisks indicate significant association (p<0.05) between PRS and level of activity.
(PDF)

S1 Table Genetic correlations. Results of genetic correlations between neuropsychiatric disorders and types of activity.
(DOCX)

S2 Table Schizophrenia PRS results. Results for all thresholds tested for association between schizophrenia PRS and level of activity. For overall activity, the beta refers to change in level of activity in standard deviations (SD) per 1SD increase in PRS. For all other types of activity, beta refers to change in minutes of activity.
(DOCX)

S3 Table Bipolar disorder PRS results. Results for all thresholds tested for association between bipolar PRS and level of activity. For overall activity, the beta refers to change in level of activity in standard deviations per 1SD increase in PRS. For all other types of activity, beta refers to change in minutes of activity.
(DOCX)

S4 Table Depression PRS results. Results for all thresholds tested for association between depression PRS and level of activity. For overall activity, the beta refers to change in level of activity in standard deviations per 1SD increase in PRS. For all other types of activity, beta refers to change in minutes of activity.
(DOCX)

S5 Table ADHD PRS results. Results for all thresholds tested for association between ADHD PRS and level of activity. For overall activity, the beta refers to change in level of activity in standard deviations per 1SD increase in PRS. For all other types of activity, beta refers to change in minutes of activity.
(DOCX)

S6 Table ASD PRS results. Results for all thresholds tested for association between ASD PRS and level of activity. For overall activity, the beta refers to change in level of activity in standard deviations per 1SD increase in PRS. For all other types of activity, beta refers to change in minutes of activity.
(DOCX)

S7 Table Full model PRS results. Results of the association between PRS and overall level of activity when co-varying for alcohol use, cannabis use, substance or behavioural addiction, smoking status, fluid intelligence, and Townsend deprivation index.
(DOCX)

S8 Table Results unadjusted for BMI. Results of the association between neuropsychiatric diagnosis or PRS and overall level of activity, without adjusting for BMI.
(DOCX)
