# Supplementary figures and images for: Association of genetic liability for psychiatric disorders with accelerometer-assessed physical activity in the UK Biobank
Source: PLoS One. 2021 Mar 26;16(3):e0249189. doi: 10.1371/journal.pone.0249189 (PMC8508577; doi:10.1371/journal.pone.0249189)

**S1 Fig. Timeline of data collection**

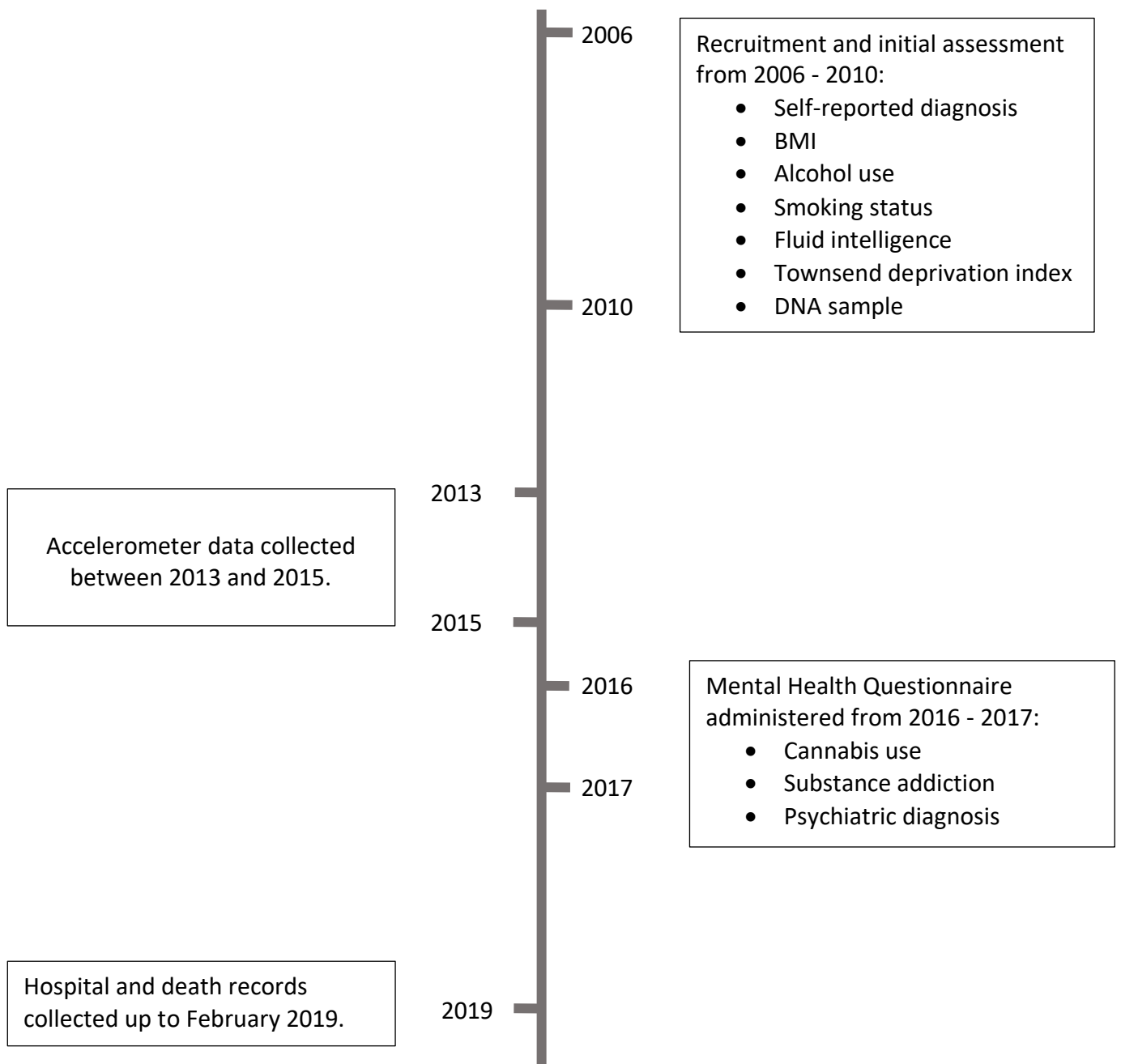

Supplement: S1 Fig — (PDF) [file pone.0249189.s001.pdf]

**S2 Fig. Flow diagram of exclusion criteria**

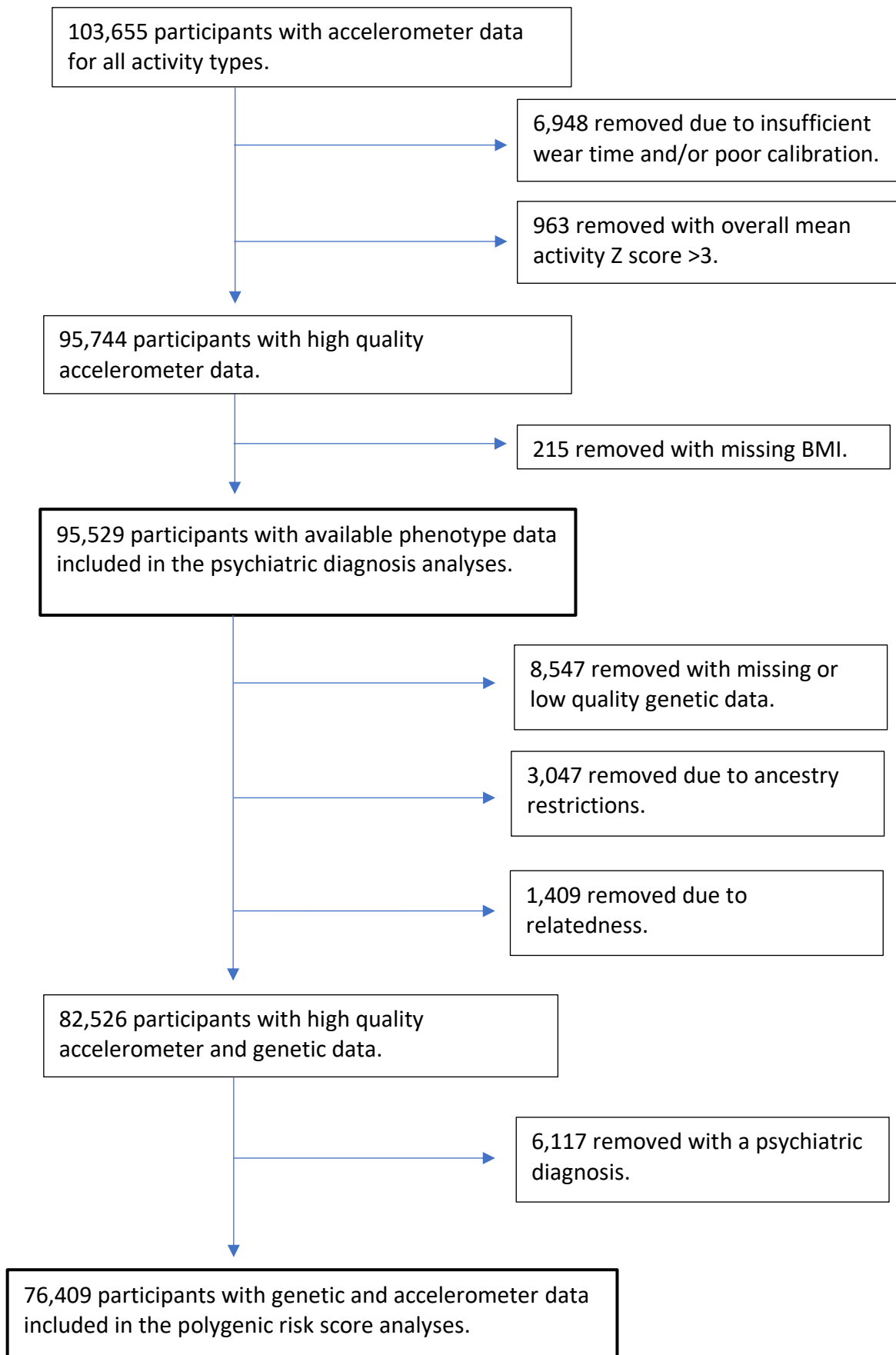

Supplement: S2 Fig — (PDF) [file pone.0249189.s002.pdf]
