# Supplementary material for: Association of genetic liability for psychiatric disorders with accelerometer-assessed physical activity in the UK Biobank
Source: PLoS One. 2021 Mar 26;16(3):e0249189. doi: 10.1371/journal.pone.0249189 (PMC8508577; doi:10.1371/journal.pone.0249189)
Supplement: S3 Fig — Proportion of variance (R2) explained by each polygenic score threshold, relative to a baseline model including only covariates, for (A) overall activity, and (B) specific types of activity. Asterisks indicate significant association (p<0.05) between PRS and level of activity. (PDF) [file pone.0249189.s003.pdf]

S3 Fig. Variance explained by polygenic risk scores

Proportion of variance (R^2) explained by each polygenic score threshold, relative to a baseline model including only covariates, for (A) overall activity, and (B) specific types of activity. Asterisks indicate significant association ($p<0.05$) between PRS and level of activity.

A

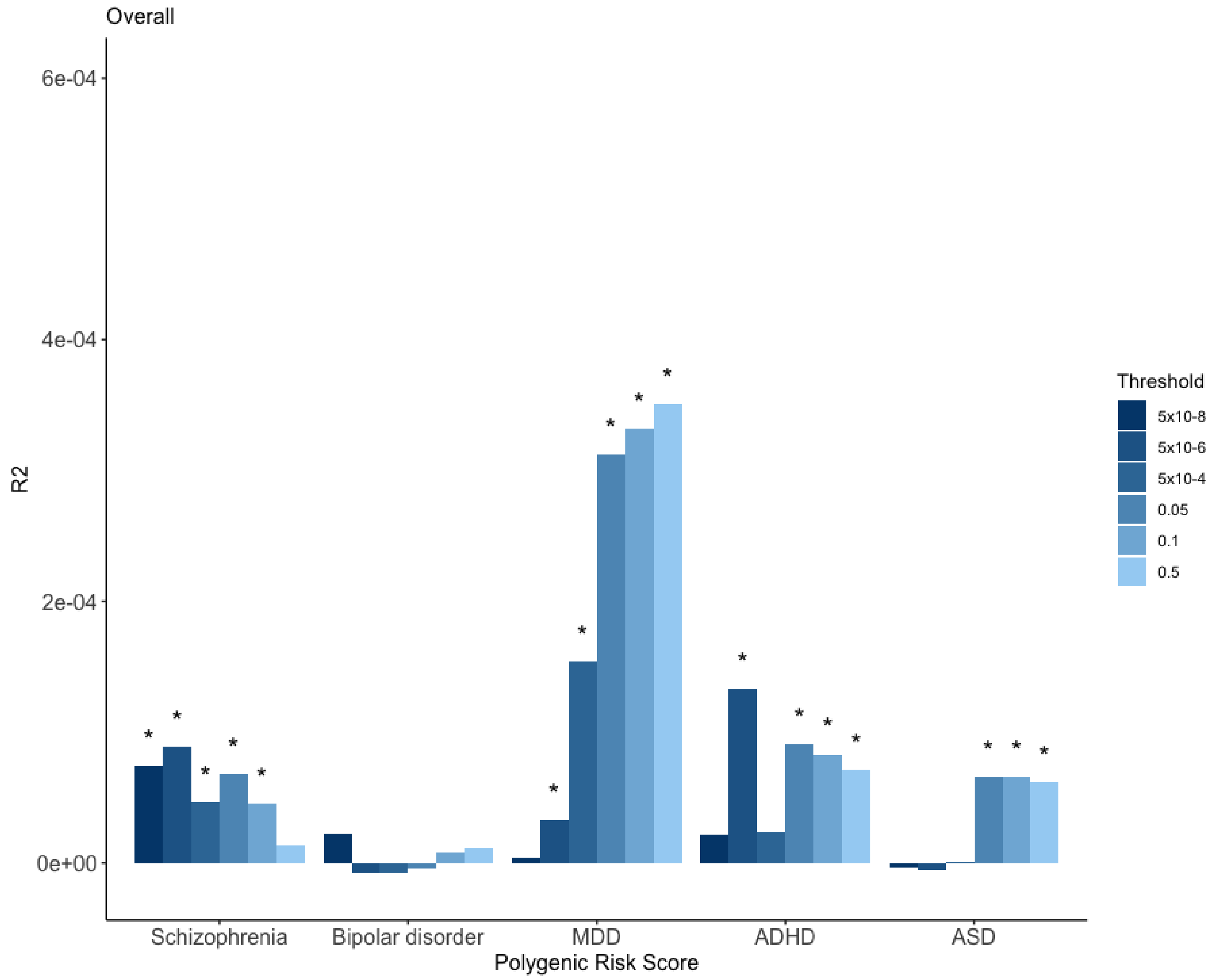

B

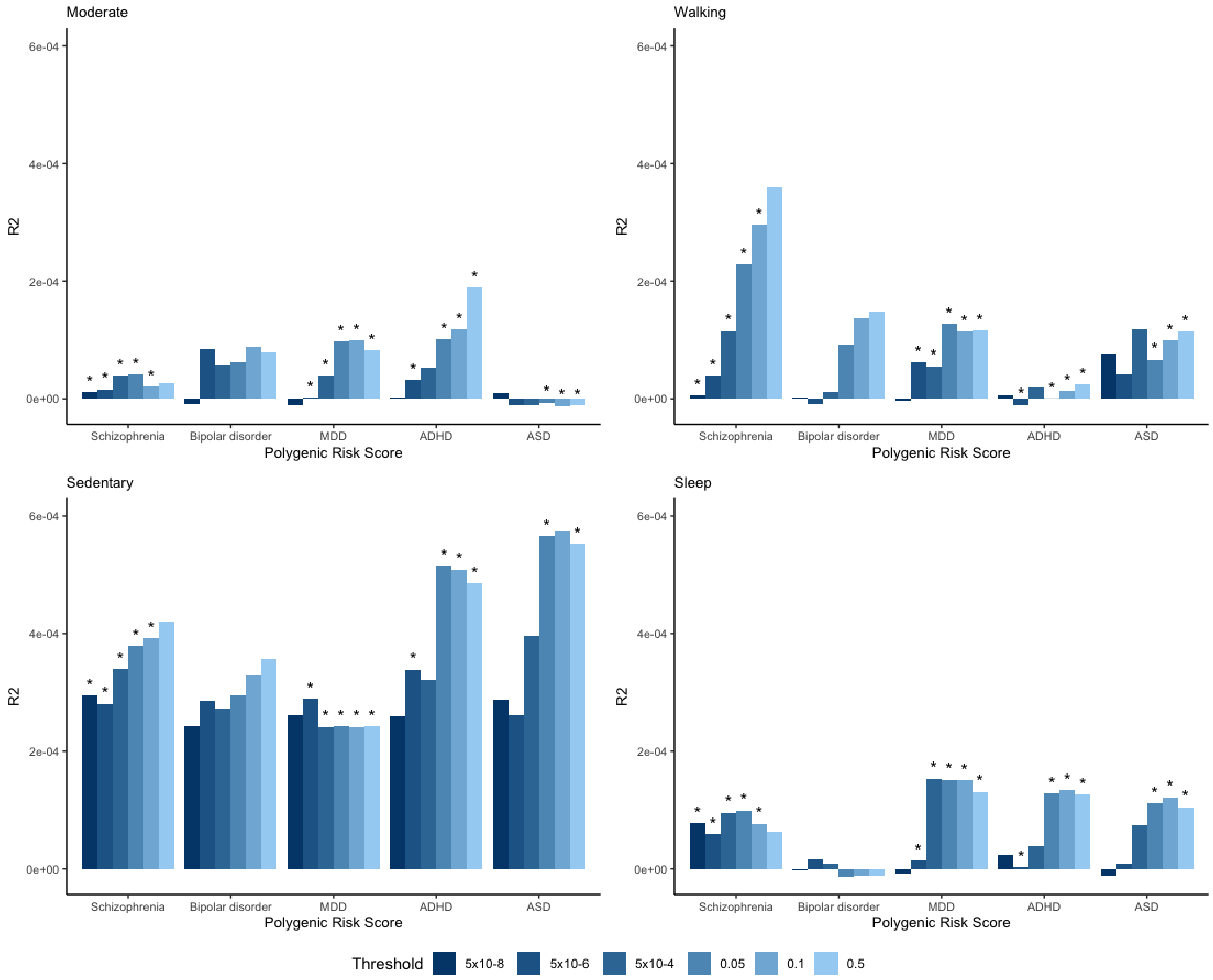

Moderate
Walking
Sedentary
Sleep
R2
6e-04
4e-04
2e-04
0e+00
Schizophrenia
Bipolar disorder
MDD
ADHD
ASD
Polygenic Risk Score
Threshold
5x10-8
5x10-6
5x10-4
0.05
0.1
0.5
