# Supplementary material for: Association of genetic liability for psychiatric disorders with accelerometer-assessed physical activity in the UK Biobank
Source: PLoS One. 2021 Mar 26;16(3):e0249189. doi: 10.1371/journal.pone.0249189 (PMC8508577; doi:10.1371/journal.pone.0249189)
Supplement: S1 Table — Results of genetic correlations between neuropsychiatric disorders and types of activity. (DOCX) [file pone.0249189.s004.docx]

## S1 Table. Genetic correlations

Results of genetic correlations between neuropsychiatric disorders and types of activity.

| Psychiatric disorder | Activity type | Genetic correlation | Standard error | P-value |
| --- | --- | --- | --- | --- |
| Schizophrenia | Overall | -0.04 | 0.03 | 0.24 |
| Schizophrenia | Moderate | 0.07 | 0.05 | 0.19 |
| Schizophrenia | Walking | 0.11 | 0.03 | 0.01 |
| Schizophrenia | Sedentary | -0.09 | 0.03 | 0.02 |
| Schizophrenia | Sleep | 0.07 | 0.03 | 0.04 |
| Bipolar disorder | Overall | 0.03 | 0.04 | 0.50 |
| Bipolar disorder | Moderate | 0.22 | 0.06 | 4x10^-3^ |
| Bipolar disorder | Walking | 0.11 | 0.04 | 0.05 |
| Bipolar disorder | Sedentary | -0.07 | 0.04 | 0.15 |
| Bipolar disorder | Sleep | <0.00 | 0.04 | 0.98 |
| Depression | Overall | -0.10 | 0.03 | 0.01 |
| Depression | Moderate | -0.01 | 0.05 | 0.98 |
| Depression | Walking | -0.10 | 0.04 | 0.02 |
| Depression | Sedentary | 0.03 | 0.04 | 0.50 |
| Depression | Sleep | 0.04 | 0.03 | 0.21 |
| ADHD | Overall | 0.08 | 0.05 | 0.15 |
| ADHD | Moderate | 0.14 | 0.07 | 0.08 |
| ADHD | Walking | <0.00 | 0.05 | 0.99 |
| ADHD | Sedentary | -0.08 | 0.05 | 0.15 |
| ADHD | Sleep | 0.01 | 0.05 | 0.98 |
| ASD | Overall | -0.11 | 0.05 | 0.05 |
| ASD | Moderate | -0.16 | 0.09 | 0.15 |
| ASD | Walking | -0.12 | 0.07 | 0.15 |
| ASD | Sedentary | 0.25 | 0.06 | 3.0x10^-3^ |
| ASD | Sleep | -0.20 | 0.06 | 0.01 |
