# Supplementary material for: Association of genetic liability for psychiatric disorders with accelerometer-assessed physical activity in the UK Biobank
Source: PLoS One. 2021 Mar 26;16(3):e0249189. doi: 10.1371/journal.pone.0249189 (PMC8508577; doi:10.1371/journal.pone.0249189)
Supplement: S6 Table — Results for all thresholds tested for association between ASD PRS and level of activity. For overall activity, the beta refers to change in level of activity in standard deviations per 1SD increase in PRS. For all other types of activity, beta refers to change in minutes of activity. (DOCX) [file pone.0249189.s009.docx]

**S6 Table. ASD PRS results**

Results for all thresholds tested for association between ASD PRS and level of activity. For overall activity, the beta refers to change in level of activity in standard deviations per 1SD increase in PRS. For all other types of activity, beta refers to change in minutes of activity.

| Activity | Threshold | beta | Lower CI | Upper CI | P-value |
| --- | --- | --- | --- | --- | --- |
| Overall | 5x10^-8^ | -0.003 | -0.01 | 0.003 | 0.40 |
|  | 5x10^-6^ | 0.002 | -0.004 | 0.01 | 0.48 |
|  | 5x10^-4^ | -0.003 | -0.01 | 0.003 | 0.31 |
|  | 0.05 | -0.01 | -0.01 | -0.002 | 0.01 |
|  | 0.1 | -0.01 | -0.01 | -0.002 | 0.01 |
|  | 0.5 | -0.01 | -0.01 | -0.002 | 0.01 |
| Moderate | 5x10^-8^ | -0.25 | -0.61 | 0.11 | 0.18 |
|  | 5x10^-6^ | -0.02 | -0.38 | 0.34 | 0.91 |
|  | 5x10^-4^ | -0.04 | -0.40 | 0.32 | 0.82 |
|  | 0.05 | -0.12 | -0.49 | 0.24 | 0.54 |
|  | 0.1 | 0.00 | -0.36 | 0.36 | 0.99 |
|  | 0.5 | 0.03 | -0.33 | 0.39 | 0.86 |
| Walking | 5x10^-8^ | -0.60 | -1.05 | -0.15 | 0.01 |
|  | 5x10^-6^ | -0.47 | -0.92 | -0.02 | 0.04 |
|  | 5x10^-4^ | -0.73 | -1.18 | -0.28 | 1.4x10^-3^ |
|  | 0.05 | -0.57 | -1.01 | -0.12 | 0.02 |
|  | 0.1 | -0.67 | -1.12 | -0.22 | 3.2x10^-3^ |
|  | 0.5 | -0.72 | -1.16 | -0.27 | 1.7x10^-3^ |
| Sedentary | 5x10^-8^ | 0.71 | -0.01 | 1.42 | 0.05 |
|  | 5x10^-6^ | 0.46 | -0.25 | 1.17 | 0.20 |
|  | 5x10^-4^ | 1.31 | 0.60 | 2.02 | 3.1x10^-4^ |
|  | 0.05 | 1.89 | 1.18 | 2.60 | 2.5x10^-6^ |
|  | 0.1 | 1.92 | 1.21 | 2.63 | 1.3x10^-7^ |
|  | 0.5 | 1.85 | 1.14 | 2.56 | 3.5x10^-7^ |
| Sleep | 5x10^-8^ | 0.05 | -0.48 | 0.57 | 0.86 |
|  | 5x10^-6^ | -0.35 | -0.87 | 0.17 | 0.19 |
|  | 5x10^-4^ | -0.70 | -1.22 | -0.17 | 0.01 |
|  | 0.05 | -0.83 | -1.35 | -0.30 | 0.01 |
|  | 0.1 | -0.86 | -1.39 | -0.34 | 1.3x10^-3^ |
|  | 0.5 | -0.80 | -1.33 | -0.28 | 2.6x10^-3^ |
