# Supplementary material for: Association of genetic liability for psychiatric disorders with accelerometer-assessed physical activity in the UK Biobank
Source: PLoS One. 2021 Mar 26;16(3):e0249189. doi: 10.1371/journal.pone.0249189 (PMC8508577; doi:10.1371/journal.pone.0249189)
Supplement: S7 Table — Results of the association between PRS and overall level of activity when co-varying for alcohol use, cannabis use, substance or behavioural addiction, smoking status, fluid intelligence, and Townsend deprivation index. (DOCX) [file pone.0249189.s010.docx]

**S7 Table. Full model PRS results**

Results of the association between PRS and overall level of activity when co-varying for alcohol use, cannabis use, substance or behavioural addiction, smoking status, fluid intelligence, and Townsend deprivation index.

| Polygenic risk score | Beta | Lower CI | Upper CI | P-value |
| --- | --- | --- | --- | --- |
| Schizophrenia | -0.01 | -0.03 | -0.001 | 0.04 |
| Bipolar disorder | 0.01 | -0.01 | 0.02 | 0.42 |
| Depression | -0.01 | -0.02 | 0.01 | 0.25 |
| ADHD | 0.01 | -0.002 | 0.02 | 0.10 |
| ASD | 0.002 | -0.01 | 0.01 | 0.80 |
