# Supplementary material for: Association of genetic liability for psychiatric disorders with accelerometer-assessed physical activity in the UK Biobank
Source: PLoS One. 2021 Mar 26;16(3):e0249189. doi: 10.1371/journal.pone.0249189 (PMC8508577; doi:10.1371/journal.pone.0249189)
Supplement: S8 Table — Results of the association between neuropsychiatric diagnosis or PRS and overall level of activity, without adjusting for BMI. (DOCX) [file pone.0249189.s011.docx]

**S8 Table. Results unadjusted for BMI**

Results of the association between neuropsychiatric diagnosis or PRS and overall level of activity, without adjusting for BMI.

| Polygenic risk score | Neuropsychiatric diagnosis | | Polygenic risk score | |
| --- | --- | --- | --- | --- |
|  | Beta (95% CI) | P-value | Beta (95% CI) | P-value |
| Schizophrenia | -0.4 (-0.6, -0.2) | 3.2x10^-6^ | -0.004 (-0.01, 0.003) | 0.26 |
| Bipolar disorder | -0.3 (-0.4, -0.3) | 2.2x10^-17^ | 0.005 (-0.001, 0.01) | 0.13 |
| Depression | -0.2 (-0.3, -0.2) | 1.2x10^-95^ | -0.03 (-0.03, -0.02) | 5.8x10^-16^ |
| ADHD | -0.1 (-0.3, 0.2) | 0.60 | -0.01 (-0.01, <0.000) | 0.06 |
| ASD | -0.4 (-0.6, -0.3) | 1.8x10^-6^ | -0.01 (-0.02, -0.003) | 2.2x10^-3^ |
